# Adolescent Survivors of Childhood Cancer: Biopsychosocial Challenges and the Transition from Survival to Quality of Life

**DOI:** 10.3390/children12080980

**Published:** 2025-07-25

**Authors:** Piotr Pawłowski, Karolina Joanna Ziętara, Natalia Zaj, Emilia Samardakiewicz-Kirol, Marzena Samardakiewicz

**Affiliations:** 1 Student Scientific Association at the Department of Psychology, Faculty of Medicine, Medical University of Lublin, 20-093 Lublin, Poland; pawlowskipiotr56@gmail.com (P.P.); natia.zaj@gmail.com (N.Z.); 2 Simulation Laboratory for Patient Safety, Department of Medical Education, Medical University of Lublin, 20-093 Lublin, Poland; emilia.samardakiewicz-kirol@umlub.pl; 3 Department of Psychology, Psychosocial Aspects of Medicine, Medical University of Lublin, 20-093 Lublin, Poland; marzena.samardakiewicz@umlub.pl

**Keywords:** childhood cancer survivor, long-term care, quality of life, psychosocial aspects, survivorship experience, adolescent health, pediatric oncology

## Abstract

Background/Objectives: The increasing population of childhood cancer survivors presents new challenges for healthcare systems worldwide. While advances in oncological treatments have dramatically improved survival rates, survivors face a broad spectrum of late effects that extend beyond the biological to encompass profound psychological and social dimensions. Methods: This quasi-systematic review synthesizes data from recent studies on adolescent survivors, revealing significant disruptions in cognitive function, mental health, social integration, education, romantic relationships, and vocational outcomes. Results: This review highlights the inadequacy of a solely biomedical model and advocates for a biopsychosocial approach to long-term follow-up care. An emphasis is placed on the necessity of personalized, interdisciplinary, and developmentally informed interventions, especially in countries like Poland, where structured survivorship care models remain underdeveloped. Conclusions: The findings underscore the importance of integrating medical, psychological, and social services to ensure adolescent cancer survivors achieve not only physical recovery but also meaningful life participation and emotional well-being.

## 1. Introduction

A childhood cancer survivor is currently defined as an individual who was diagnosed with cancer during childhood and has successfully completed oncological treatment. In the 21st century, advances in medical science have led to significant improvements in cancer treatment outcomes, contributing to a steadily increasing population of long-term survivors with the prospect of an extended life expectancy. However, contemporary anticancer therapies, while effective in improving survival, are associated with a wide range of adverse effects, including late-onset complications that may emerge months or even years after the completion of treatment [1,2].

The biopsychosocial perspective in pediatric oncology emphasizes the interplay of biological (e.g., treatment-related sequelae), psychological (e.g., anxiety, post-traumatic stress), and social (e.g., peer reintegration difficulties) factors in the long-term well-being of childhood cancer survivors. This framework aligns with the WHO’s definition of health as “a state of complete physical, mental and social well-being and not merely the absence of disease or infirmity” (WHO, 1948) [3]. Consequently, survivorship care must be interdisciplinary, addressing medical, psychological, and social dimensions to promote a holistic recovery [4].

These late effects are diverse in nature and may impact various organ systems, cognitive and psychological functioning, as well as social integration. Their manifestation is influenced by numerous factors, including the type and intensity of the treatment received, the age at diagnosis, the lifestyle, and the individual’s psychological, environmental, and socio-economic circumstances. As a result, there is a growing consensus among experts that survivorship care must be long-term, personalized, and multidisciplinary in nature [5,6].

Studies conducted in the United States have highlighted the challenges childhood cancer survivors face in their transition to adulthood. Compared to their healthy peers, survivors often experience a more difficult start to adult life. They tend to perform worse academically, achieve lower levels of education, face greater difficulties in choosing a career path and securing stable employment, and encounter obstacles in forming intimate relationships and maintaining social connections. These challenges can lead to social isolation, a reduced quality of life, and unmet healthcare needs [7,8].

In response, several countries have developed survivorship care models that prioritize coordinated, interprofessional support through long-term follow-up (LTFU) care programs. These programs are designed to monitor the health status, detect and manage late effects, and provide psychosocial support throughout the survivor’s lifespan. The effectiveness of such models is largely dependent on the clinical experience of the care team, the quality of the care coordination, and the efficiency of interdisciplinary communication [9,10].

Despite the growing need, Poland currently lacks a standardized and cohesive national system for the follow-up care of childhood cancer survivors. The duration and scope of post-treatment surveillance vary significantly between treatment centers, frequently relying on informal protocols rather than evidence-based frameworks. Several systemic barriers impede the development of survivorship care, including insufficient national funding allocations, the absence of centralized survivorship registries, and a lack of policy directives from the Ministry of Health mandating LTFU. Moreover, there is a shortage of trained psycho-oncology professionals and no integration of survivorship care into primary care structures. Psychosocial services, when available, are typically provided only during active treatment, with no continuity into survivorship. Additionally, health data silos prevent coordinated care between pediatric and adult services, severely hampering transitional care efforts [11,12,13].

Specifically, this study seeks to assess the current state of the medical, psychological, and social support available to this population; examine the extent and effectiveness of existing survivorship care practices; and propose evidence-informed recommendations for the development of a coordinated, multidisciplinary model of long-term care tailored to the needs of childhood cancer survivors. The innovative nature of this article lies in its holistic approach to analysis, incorporating medical, psychological, and social perspectives and integrating findings from various fields often considered separately. This review adds a novel perspective by integrating developmental theory with recent international findings and region-specific data from Central and Eastern Europe, an area often underrepresented in psychosocial survivorship research.

## 2. Materials and Methods

Given the complexity and interdisciplinary nature of the topic, a quasi-systematic review of the literature was chosen as the methodological approach. This review combines elements of systematic review processes with greater flexibility in the selection and analysis of sources, allowing for a more comprehensive exploration of the research landscape. The quasi-systematic approach enabled the inclusion of a wide range of scientific publications, including those from outside strictly medical databases, which was crucial for providing a holistic understanding of the topic.

It is important to note that this review was not registered in any systematic review registry, such as PROSPERO, due to its exploratory nature and the absence of an initial intention to formalize the process in alignment with guidelines like PRISMA. This decision reflects the dynamic and adaptive approach of this review, which prioritized the inclusivity of diverse research sources and the development of insights based on emerging evidence, rather than adhering strictly to predefined protocols. Furthermore, this article did not conduct an assessment of the risk of systematic bias using the Cochrane Collaboration tool.

### 2.1. Databases Searched

A comprehensive literature search was conducted between February and April 2025 using the following electronic databases: (1) PubMed/MEDLINE; (2) Scopus; (3) Web of Science; (4) CINAHL (Cumulative Index to Nursing and Allied Health Literature); and (5) Google Scholar (for gray literature and reports from NGOs).

Additionally, relevant Polish-language sources were screened using (1) Polska Bibliografia Lekarska (PBL); (2) institutional repositories of leading Polish oncology and pediatric hospitals; and (3) reports from non-governmental organizations (e.g., Fundacja “Na Ratunek Dzieciom z Chorobą Nowotworową”).

### 2.2. Search Strategy and Keywords

The search strategy combined both controlled vocabulary (Medical Subject Headings—MeSH) and free-text keywords to maximize the retrieval of the relevant literature. Boolean operators (AND, OR, NOT) and truncation symbols were used to structure search queries. Search queries were as follows (PubMed/MeSH): (“Neoplasms” [MeSH] OR “cancer”) AND (“Survivors” [MeSH] OR “Cancer Survivor*” OR “Childhood Cancer Survivor*”) AND (“Long-Term Care” [MeSH] OR “long-term follow-up” OR “LTFU”) AND (“Adolescent” [MeSH] OR “Young Adult” [MeSH] OR “teenager*” OR “youth*”) AND (“Biopsychosocial” [MeSH] OR “psychosocial” [MeSH] OR “quality of life” [MeSH] OR “mental health”).

Free-text keywords were as follows: childhood cancer survivors, long-term follow-up, late effects, adolescent and young adult (AYA) survivors, psychosocial challenges, biopsychosocial model, survivorship care plan, healthcare coordination, and transition to adulthood.

### 2.3. Inclusion and Exclusion Criteria

Inclusion criteria

(1) Publication Date Range: Only studies published between 2015 and 2025 will be included to ensure that this review is based on the most current research, reflecting recent advancements in cancer treatment and the evolving understanding of childhood cancer survivorship.
(2) Types of Publications
  - Peer-reviewed original research articles: Studies that provide empirical data and detailed results on biopsychosocial outcomes, healthcare needs, and the long-term effects of cancer in childhood survivors.
  - Systematic reviews and meta-analyses: These sources are valuable for synthesizing evidence from multiple studies, providing higher-level insights into trends and gaps in the research.
  - Guidelines and expert consensus documents: In addition to clinical guidelines, documents from international health organizations or cancer advocacy groups will be considered if they address best practices or new frameworks for care specific to childhood cancer survivors.
(3) Gray Literature
  Reports, white papers, and technical papers: The relevant gray literature will be included if published by reputable institutions, such as universities, governmental health agencies, or established NGOs. These materials often include reports on current health policies, strategies for managing childhood cancer survivorship, or the evaluations of care models that may not be published in traditional peer-reviewed journals.
  Policy documents and recommendations: Publications from health organizations, international agencies (such as the WHO), or advocacy groups that provide recommendations or policy guidance for the care of childhood cancer survivors, especially regarding long-term follow-up care, psychosocial support, and healthcare infrastructure.
(4) Language: Publications must be in English or Polish to ensure the proper evaluation of the content and relevance of the research for the target population.
(5) Study Focus:
  Studies must address one or more of the following:
  - Biopsychosocial outcomes: Long-term physical, psychological, and social effects of childhood cancer and its treatments.
  - Late effects: Chronic or delayed effects resulting from cancer treatments, including physical health problems, cognitive impairments, or emotional challenges.
  - LTFU (long-term follow-up) models: Models of care that ensure ongoing monitoring and support for childhood cancer survivors, especially as they transition into adolescence or adulthood.
  - Healthcare needs: An exploration of the healthcare requirements, including specialized care, psychosocial support, and rehabilitation services, for childhood cancer survivors as they age.

Exclusion criteria:

(1) Studies not involving childhood or adolescent cancer survivors:
  Excludes studies focusing on adult cancer survivors or those not involving individuals diagnosed before 18 years of age.
(2) Articles focused exclusively on acute treatment or end-of-life care:
  Excludes studies that focus only on cancer treatment phases or palliative care, rather than long-term survivorship outcomes.
(3) Opinion pieces or editorials without empirical basis:
  Excludes opinion articles, editorials, and commentaries that lack scientific data or empirical research.
(4) Duplicate publications:
  Excludes studies that are published multiple times with the same data, including abstracts and full-text versions.
(5) Studies lacking clear methodological rigor:
  Excludes studies that do not provide detailed methodological information, such as sample size or data analysis methods.
(6) Studies with insufficient or non-representative sample populations:
  Excludes studies with small sample sizes or non-representative populations, limiting generalizability.
(7) Studies without clear outcomes related to survivorship:
  Excludes studies that do not focus on long-term outcomes, healthcare needs, or biopsychosocial impacts of childhood cancer survivorship.
(8) Studies published in non-peer-reviewed sources:
  Excludes publications that have not undergone peer review, ensuring scientific rigor.
(9) Studies with significant methodological flaws or high risk of bias:
  Excludes studies with major methodological flaws or high risk of bias that could distort results.
(10) Studies focusing on non-medical aspects unrelated to health outcomes:
  Excludes studies focusing on entertainment, lifestyle choices, or unrelated topics without relevance to health outcomes.
(11) Studies without full-text availability:
  Excludes studies that are not available in full text, ensuring access to complete data for evaluation.

### 2.4. Study Selection and Data Extraction

Two independent reviewers conducted title and abstract screening based on the eligibility criteria. Full texts of the selected articles were then assessed for final inclusion. A standardized data extraction form was used to collect information on the following: (1) study design and setting; (2) population characteristics (e.g., age, type of cancer, time since treatment); (3) reported outcomes (medical, psychological, social); (4) type and structure of LTFU care; and (5) key findings and recommendations. To ensure consistency and reduce bias, regular communication between the reviewers was encouraged throughout the process. In cases of disagreement, the reviewers aimed to engage in constructive discussions to reach a consensus. When this was not possible, a third reviewer was consulted to provide an independent judgment, allowing for an unbiased resolution. This approach ensured that the final decisions were based on a comprehensive evaluation and that the review process remained transparent and rigorous.

### 2.5. Quasi-Systematic Flow Diagram

**Figure 1:**
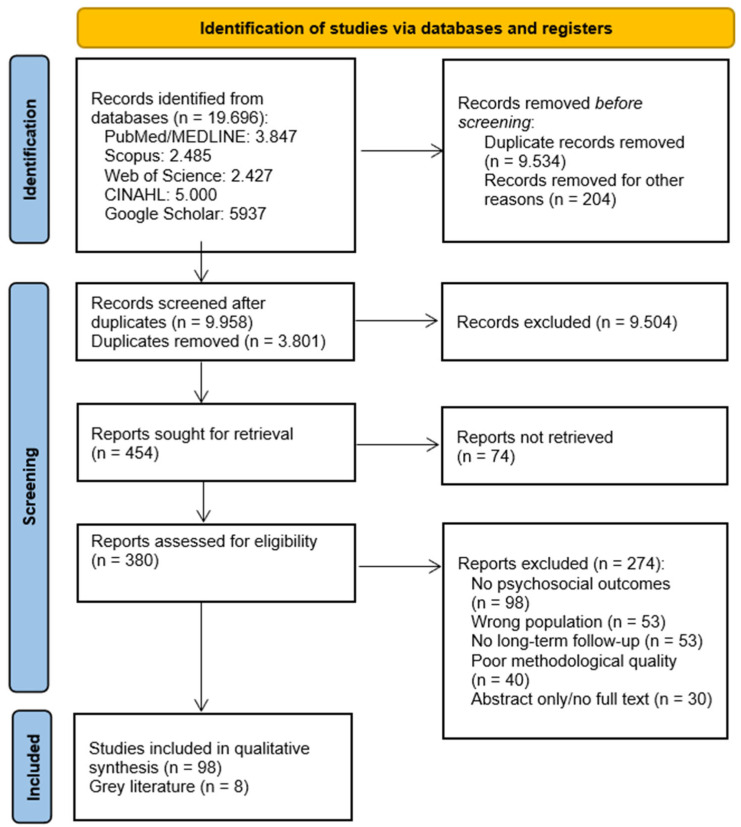
A summary of the article selection process for the quasi-systematic review [14].

Quantitative search data is provided in the quasi-systematic flow diagram, considering the limitations described in the methodology section above (Figure 1).

## 3. Results

### 3.1. The Biological Aspect of the Health of Adolescent Childhood Cancer Survivors

As previously mentioned, the remarkable advances in anticancer therapies for childhood malignancies—including chemotherapy, radiotherapy, immunotherapy, and surgical interventions—have significantly improved survival rates among pediatric patients. However, the effectiveness of these treatments is often unfortunately correlated with the occurrence of biological complications, which may manifest both during the therapy and many years after its completion (so-called long-term complications). These complications are typically multiorgan in nature, and their etiopathogenesis depends on several factors, such as the type of therapy used, the cumulative dose, the age of the child at treatment, and, most importantly, the individual susceptibility of the patient’s organism [15].

International experts, based on clinical and research evidence, have classified these complications into several major categories, focusing on organ-specific damage. These include cardiotoxicity, nephrotoxicity and renal dysfunction, hepatotoxicity, endocrine disorders, growth disturbances and skeletal–muscular developmental abnormalities, neurotoxicity and cognitive impairments, immunosuppression and immune dysregulation, and secondary malignant neoplasms [15,16].

To clarify and systematize this group of anticancer therapy-related complications, Table 1 presents a summary of the biological complications described in the scientific literature. The biological condition of childhood cancer survivors, with an emphasis on their pathophysiological mechanisms, is detailed in the Supplementary Material accompanying this study. Surgical-treatment-related complications were not included in the present analysis, as they are generally consistent with and analogous to outcomes observed following surgical procedures performed for non-oncologic indications.

### 3.2. Psychological and Social Health Aspects of Adolescent Childhood Cancer Survivors

#### 3.2.1. Scale of Psychological Burden

Current survivorship guidelines from the International Late Effects of Childhood Cancer Guideline Harmonization Group, the Children’s Oncology Group Long-Term Follow-Up Guidelines, and NCCN Survivorship v2.2024 recommend that every follow-up visit for adolescent survivors (13–19 years) includes a screening for depression, anxiety, and post-traumatic stress disorder (PTSD) with validated tools such as PHQ-9, GAD-7, and UCLA PTSD-RI; higher-risk patients (e.g., central nervous system tumors, prior cranial radiotherapy, chronic pain) require more frequent assessments (12- to 24-month intervals or sooner). The COG definition of psychological distress comprises major depressive disorder, generalized anxiety disorder, full-syndrome PTSD, suicidal ideation, and severe adjustment disorders, and it suggests the BSI-18 or PROMIS-29 when diagnostic clarity is needed [56,57].

The pooled evidence illustrates the magnitude of the problem. A meta-analysis by Osmani et al. of >15,000 adolescent and young adult survivors reported prevalence rates of 32% for global distress, 29% for anxiety, and 24% for depression, corroborating findings from the Childhood Cancer Survivor Study (CCSS), where depression and anxiety remained significantly more common than in sibling controls (OR 1.55 and 2.00, respectively) [58]. The risk of full-criteria PTSD is likewise elevated more than two-fold (RR 2.36, 95% CI 1.37–4.06), particularly after CNS tumors or cranial irradiation. Suicidal ideation affects roughly 9% of survivors (RR 1.67), although completed suicide remains uncommon (0.30%; RR 1.50, non-significant) [59].

Cohort data from the CCSS (median age about 17 years) demonstrate markedly higher rates of depression (11.7%) and anxiety (7.4%) among survivors diagnosed between 11 and 21 years of age compared with their siblings (OR = 1.55 and 2.00, respectively). Roughly 16% of older adolescents also report a clinically significant fear of cancer recurrence (FCR), a concern that intensifies in the presence of chronic neurological sequelae or unemployment [60,61]. Complementary Swiss longitudinal data (median age 16 years) reveal that the prevalence of global distress rises from 13% to 25% over a 2.4-year interval—an effect most pronounced in females and survivors of CNS tumors. Importantly, the co-occurrence of late somatic complications, such as cardiomyopathy, endocrinopathies, or chronic pain, nearly doubles the odds of depressive–anxiety disorders, a pattern documented both in the CCSS and in other population-based analyses [61,62,63].

Despite this substantial burden, Central and Eastern Europe still lack prospective, adolescent-focused cohort studies, a gap highlighted by IGHG experts and reiterated by the Swiss investigators. This shortfall underscores the urgent need for harmonized registries and standardized screening protocols across the 13–19 year age range [56,62]. The most common mental health disturbances among adolescent childhood cancer survivors are summarized in Table 2.

Despite the growing population of childhood cancer survivors in Poland, published data on psychosocial outcomes remain scarce. Existing reports, including the 2023 national report “*Życie po nowotworze*”, indicate that access to psychological care during follow-up is limited and often fragmented, with no national standards for monitoring depression, anxiety, or post-traumatic stress symptoms. Survivorship clinics are rare, and psychological screening tools such as PHQ-9 or GAD-7 are seldom used in routine follow-up, especially beyond tertiary care settings. Moreover, educational and vocational counseling is inconsistently available. These systemic gaps underscore the need for localized research and the integration of psychosocial assessments into long-term care protocols in Poland. The lack of registry-based studies or longitudinal cohorts limits the ability to benchmark the psychosocial risk among Polish survivors relative to international standards [9,66].

#### 3.2.2. Neurocognitive Impairments in Adolescent Survivors

Neurocognitive late effects—persistent deficits in attention, processing speeds, memory, and executive control that arise after central nervous system-directed or high-dose systemic therapy—rank among the most frequent sequelae of childhood cancer. In a prospective St. Jude Total Therapy XV/XVI cohort, 41% of adolescent survivors of acute lymphoblastic leukemia (ALL) performed ≥1 SD below age-adjusted norms in at least one cognitive domain. Comparable but even higher prevalences were observed in the multi-center CCSS: 53% of ALL survivors and 69% of central nervous system (CNS) tumor survivors met the same ≤−1 SD criterion, translating into odds ratios of 3.1 and 5.6, respectively, when benchmarked against sibling controls [67,68,69].

The therapy intensity is the primary predictor of neurocognitive outcomes. Within the St. Jude series, higher cumulative intravenous methotrexate and cranial irradiation ≥18 Gy were independently associated with a lower full-scale IQ and a slower processing speed. Modern proton beam therapy reduces but does not eliminate this risk: in a matched analysis of pediatric medulloblastoma, proton-treated survivors achieved mean processing speed z-scores significantly higher than photon-treated controls, yet their scores remained below population norms [67,70].

Longitudinal evidence indicates that neurocognitive deterioration rarely plateaus. In a seminal St. Jude series of 71 medulloblastoma survivors, the full-scale IQ fell by a mean of 1.5 points per year during the first five years after cranial irradiation, with the steepest decline in children treated before 7 years of age. A subsequent dose–response meta-analysis of 14 longitudinal cohorts confirmed a cumulative effect, demonstrating that each additional gray delivered to the temporal lobe was associated with an extra 0.05 point annual IQ loss [71,72].

Current survivorship guidelines, COG LTFU v6.0 (2023), and the IGHG neurocognitive harmonization document (2024) recommend baseline neuropsychological testing (WISC-V or WAIS-IV core subtests, CPT-3, BRIEF-2) within 12 months of therapy completion and a re-evaluation every 2–3 years [56,57].

In a pilot study of 45 survivors (58% ALL, 20% CNS tumors), the computerized CogState Composite required a mean 26 min to complete versus 42 min for a conventional battery (about 38% faster) and achieved a sensitivity of 0.47 and a specificity of 0.84 for detecting an academic deficit of ≥1 SD. A randomized trial involving 158 adolescents showed that an eight-week CogMed working memory program increased digit span scores by +0.34 SD and academic fluency by +0.27 SD at the six-month follow-up. Pharmacological augmentation has also been explored: in a double-blind crossover study of 83 survivors, a single dose of methylphenidate 0.6 mg kg^−1^ raised the Processing Speed Index by about four points relative to the placebo [73,74,75].

In Poland, neuropsychological monitoring is not routinely embedded in survivorship care, which may delay the detection of cognitive issues. A 2022 study conducted in Kraków using P300 event-related potential screening among childhood ALL survivors revealed a prolonged latency and a reduced amplitude in approximately one quarter of patients, exposing subclinical deficits not captured by routine care. These findings support the integration of objective neurophysiological tools alongside psychometric tests to enhance the early identification of cognitive decline [76].

#### 3.2.3. Peer and Psychosocial Development

Adolescence is characterized by complex developmental transitions that involve the consolidation of identity, the formation of intimate relationships, and increasing autonomy. According to Erikson’s psychosocial theory, this period centers on the task of resolving identity versus role confusion, a process essential for developing a stable sense of self. Arnett’s theory of emerging adulthood further expands this framework by emphasizing continued identity exploration and the gradual assumption of adult roles between ages 18 and 25. These models provide a conceptual foundation for understanding how chronic illness or early medical trauma—such as childhood cancer—can disrupt normative psychosocial development and delay the acquisition of key milestones like autonomy, intimacy, and role fulfillment [77,78].

Adolescence is a critical period marked by achieving autonomy, forming a stable body image, forging peer and romantic relationships, and planning educational and vocational trajectories. Childhood cancer survivors must navigate these developmental milestones under the shadow of late-appearing physical and neurocognitive effects, which amplify their psychosocial vulnerability to emotional and social disruption [79,80].

A longitudinal cohort study conducted within the Dutch Childhood Cancer Survivor Study (DCCSS-LATER) cohort revealed that young adult survivors of childhood CNS tumors often experience marked difficulties in fulfilling age-appropriate psychosocial developmental tasks. Survivors aged 18–30 years, several years after treatment completion, were significantly more likely to lag behind their healthy peers in forming independent identities, establishing emotional and physical intimacy, and assuming adult roles such as self-directed decision-making and romantic attachment. These developmental setbacks are not merely incidental but appear to reflect persistent disruptions in social and psychological maturation linked to early medical trauma and the neurocognitive burden. Quantitatively, this was reflected in significantly reduced scores on autonomy (Cohen’s d = −0.36) and psychosexual development (d = −0.46) in survivors compared with age-matched controls. Furthermore, their likelihood of reaching normative developmental milestones in these domains was found to be substantially reduced, with odds ratios ranging from 0.25 to 0.48 (*p* < 0.001). In addition, survivors consistently reported fewer meaningful friendships, limited participation in social activities, and more frequent experiences of exclusion or isolation from peer groups (OR = 0.23–0.47) [81].

Survivors often replace or supplement peer support with a closer reliance on family, particularly parental figures. This divergence from normative adolescent development, where peer affiliation plays a central role in shaping identity, autonomy, and emotional regulation, can significantly impede survivors’ ability to develop age-appropriate social competencies. Unlike their healthy counterparts, who increasingly draw on friendships for emotional expression and decision-making, many survivors maintain dependency patterns rooted in the caregiver dynamic from their treatment years. A systematic review by Hemming et al. highlights that this sustained reliance on family networks, while offering short-term emotional stability, may delay the development of independent social functioning and reduce resilience in unfamiliar social environments. The review further notes that diminished peer contact can compromise survivors’ confidence in navigating interpersonal conflict, forming romantic attachments, and asserting boundary competencies typically cultivated during adolescence in peer contexts [80].

Educational reintegration poses another subtle yet impactful barrier to psychosocial development. While high school graduation rates among childhood cancer survivors generally remain comparable to the general population, many survivors must rely on academic accommodations, such as individualized education plans (IEPs), reduced course loads, or extended times for exams. Though these interventions support cognitive and physical recovery, they can also isolate students from mainstream classroom experiences, inadvertently limiting their social engagement with peers. According to Milner et al., such structural adjustments, while necessary, may reduce a survivor’s sense of inclusion and connection within the educational setting. This is particularly significant given the central role of schools as social arenas during adolescence. Feelings of separateness, reduced participation in extracurricular activities, and stigmatization linked to perceived “special treatment” have all been cited as barriers to social integration. Over time, these factors may contribute to increased social withdrawal and perceived difference, compounding emotional vulnerability and delaying developmental transitions [82].

Psychosexual development, another cornerstone of adolescent maturation, is frequently disrupted among survivors. In the DCCSS-LATER 2 study, delays in initiating romantic and sexual relationships were particularly prominent in individuals aged 18–24 years, many of whom had minimal or no experience with dating or physical intimacy well into early adulthood. A notable 44.8% of participants reported feeling insecure about their physical appearance, citing treatment-related changes such as scarring, hair loss, weight fluctuation, and delayed puberty as major barriers to intimacy. These insecurities were not merely cosmetic concerns but were deeply linked to survivors’ perceptions of desirability, bodily ownership, and sexual self-worth. For many, the internalized belief of being “different” or “damaged” hindered both the initiation and maintenance of romantic relationships. Furthermore, survivors expressed apprehension about disclosing their cancer history to potential partners, fearing pity, rejection, or being perceived as high-risk in terms of fertility or long-term health. Such challenges underscore the profound, multidimensional nature of psychosexual late effects encompassing not only physical sequelae but also emotional, relational, and existential dimensions of survivorship [83].

Concerns about fertility are similarly prevalent. In a Canadian cohort study of 311 long-term childhood cancer survivors (mean age 22.7 years), 21.2% reported fertility-related concerns at a single time point. Among a subsample of 80 individuals who completed all three waves of assessment, 30% expressed such concerns at least once, while 9% reported persistent, ongoing anxiety related to fertility. These findings highlight the importance of integrating reproductive health counseling and psychosocial support into long-term survivorship care [84].

In Poland, many survivors lack access to structured peer support or reintegration initiatives, increasing their risk of social isolation. National data indicate that over 40% of childhood cancer survivors report long-term difficulties establishing or maintaining peer relationships, often due to inconsistent school-based support and the absence of formal psychosocial interventions. Moreover, a clinical study conducted among adolescent survivors revealed that more than one-third experience persistent feelings of loneliness and emotional distress up to five years post-treatment, underscoring the critical importance of sustained psychosocial follow-up. These findings emphasize the need to integrate formal psychosocial and peer support programs into survivorship care pathways to support social functioning and emotional well-being [13,66].

Access to professional psychosocial care in Poland remains uneven: while large cancer centers increasingly offer psycho-oncology services, many smaller or regional centers do not employ any psycho-oncologists, leaving survivors without access to specialized support. There is currently no unified national standard mandating psychosocial monitoring in survivorship protocols, resulting in substantial regional variability. Combined with the high rates of social withdrawal reported earlier, these systemic deficiencies underscore the need for structured psychosocial and peer support programs integrated into survivorship care across the country [85,86].

Childhood cancer survivors, especially those treated for CNS tumors, face significant challenges in achieving autonomy, peer-related integration, psychosexual development, and managing fertility concerns. Their reliance on familial support, combined with persistent social isolation and self-image struggles, underscores the necessity of tailored programs. Structured psychosocial monitoring, alongside peer-based interventions and comprehensive fertility counseling, is paramount for supporting identity formation, interpersonal skills, and a successful transition to adulthood.

#### 3.2.4. Risk, Resilience, and Screening in Psychosocial Survivorship

The psychosocial outcomes of adolescent cancer survivors are shaped not only by their diagnosis and treatment but also by a complex interplay of psychological and environmental factors. A growing body of evidence shows that how young survivors perceive their illness, as well as the quality of their social environment, significantly influences the long-term adjustment. For example, survivors who internalize negative illness beliefs (e.g., “I am still damaged”) or experience low self-worth and poor social connectedness are more likely to develop symptoms of anxiety, depression, and a reduced quality of life. These associations were demonstrated in a large Dutch study, where survivors with maladaptive cognitive–emotional profiles had significantly worse psychosocial outcomes across multiple domains [81].

Conversely, protective factors such as strong family support, adaptive coping, and high self-efficacy appear to buffer the negative impact of early life illness. In the same cohort, those who reported feeling understood and emotionally supported by family or peers had a higher quality of life and were more likely to reach developmental goals in education, work, and relationships [81,87].

Recognizing the central role of modifiable psychosocial factors has led to an increased emphasis on structured monitoring within survivorship care. Leading guidelines from the COG and the IGHG recommend systematic psychosocial screening at least every 12–24 months during adolescence—or more frequently in high-risk groups (e.g., survivors of CNS tumors, those with chronic pain or prior psychological symptoms) [56,57].

Standardized psychosocial and neurocognitive screening is a key component of survivorship care for adolescent and young adult (AYA) childhood cancer survivors. Given the elevated risk of emotional distress, cognitive dysfunction, and social withdrawal observed in this population, brief and validated tools are essential for an early detection and appropriate interventions. Among the most widely recommended is the Patient Health Questionnaire-9 (PHQ-9), a nine-item self-report scale used to identify depressive symptoms. In a large-scale meta-analysis, Levis et al. demonstrated its diagnostic accuracy, reporting a pooled sensitivity of approximately 80% and a specificity of 85% for detecting major depressive disorder. Importantly, studies in pediatric oncology settings have shown that the PHQ-9 can more reliably capture depressive episodes among survivors than among age-matched peers from the general population [88,89].

Similarly, the Generalized Anxiety Disorder-7 (GAD-7) scale has proven highly effective for anxiety screening in adolescent cohorts. Its internal consistency (Cronbach’s α about 0.92) and strong test–retest reliability make it a practical tool in follow-up contexts, including oncology outpatient settings. In survivors, the early identification of anxiety symptoms is critical to prevent escalation and long-term impairment in educational or relational functioning [90].

For those exposed to severe medical trauma, such as intensive chemotherapy, ICU stays, or CNS-directed treatments, the UCLA PTSD Reaction Index (PTSD-RI) is recommended by both the Children’s Oncology Group and the International Late Effects Guideline Harmonization Group. These guidelines explicitly include the PTSD-RI in screening protocols for survivors aged 13–19 years, emphasizing its clinical relevance in identifying trauma-related symptoms that may otherwise be masked by somatic complaints or academic decline [56,57].

The assessment of neurocognitive late effects is equally crucial, particularly in survivors who have undergone CNS-directed therapy. The Behavior Rating Inventory of Executive Function (BRIEF), a parent-report questionnaire, has been validated within pediatric oncology to detect real-world executive dysfunction. In a large cross-sectional study by Viola et al. involving 256 survivors of childhood ALL, the BRIEF Global Executive Composite (GEC) and Behavioral Regulation Index (BRI) demonstrated a high diagnostic specificity (87.7–89.3%) but a relatively low sensitivity (24.1–39.1%) [GEC OR 4.46 (95% CI: 1.77–11.22); BRI OR 4.33 (95% CI: 1.72–10.9)]. This suggests that while the BRIEF is effective in identifying survivors with clinically meaningful executive impairments—such as those requiring special educational services or exhibiting symptoms of attention deficit hyperactivity disorder (ADHD)—it may underdetect more subtle deficits in cognitive regulation [91,92,93].

Incorporating validated screening tools such as the PHQ-9, GAD-7, PTSD-RI, and BRIEF into survivorship care allows for the early detection of psychosocial and cognitive challenges in adolescent cancer survivors. Their routine use supports timely interventions and enhances the quality and continuity of individualized LTFU. In Poland, systematic psychosocial risk screening for adolescent cancer survivors is not part of national survivorship protocols. No recent Polish studies have evaluated the structured use of validated screening tools—such as the Distress Thermometer or the Pediatric Symptom Checklist—for identifying emotional or social risks during follow-up. Available data suggest that psychosocial assessments are conducted inconsistently and are often limited to large academic centers or research settings. This lack of standardized screening hinders the early identification of at-risk individuals and delays targeted interventions to support resilience. Implementing a national framework for psychosocial surveillance remains a critical gap in survivorship care in Poland [94].

#### 3.2.5. Education, Intimate Life, and Peer Belonging in Adolescent Cancer Survivorship

Returning to school after cancer treatment poses persistent academic and social challenges. Survivors, particularly those treated for CNS malignancies, commonly experience difficulties with attention, fatigue, and memory, which can impair school performance. According to Barrera et al., 21% of childhood cancer survivors repeated a grade, and 19% required special education services significantly more than their healthy peers (9% and 7%, respectively). To address these barriers, integrative reviews of school reintegration highlight the need for structured, multidisciplinary support. Castleberry et al., in their comprehensive analysis of post-cancer educational experiences, emphasized that close collaboration between healthcare providers, educators, and families plays a crucial role in successful reintegration. Support strategies such as tailored communication, individualized learning plans, and teacher training were associated with an improved school attendance and better emotional adjustment. These findings underscore the importance of proactive, coordinated school-based interventions as a central element of survivorship care [95,96]. In Poland, educational support for childhood cancer survivors is frequently limited to informal accommodations, such as extended deadlines or part-time attendance, without a structured educational plan. A 2021 Polish study identified that over 80% of pediatric cancer survivors reported feelings of isolation, and 42% reported negative experiences related to schooling, including difficulties with reintegration and disrupted learning patterns. These disruptions in the education process can exacerbate academic regression and impact long-term self-esteem. The findings highlight the need for formal collaboration between healthcare providers, schools, and families to create targeted educational support following treatment [97].

Building and maintaining romantic relationships can be particularly challenging for adolescent and young adult survivors of childhood cancer. The psychological and physical aftermath of treatment often interferes with intimacy, emotional openness, and self-image—core elements of relationship formation during youth. Many survivors report difficulties related to body confidence, scars, delayed sexual development, or concerns about attractiveness, which in turn can delay the initiation of romantic partnerships or lead to the avoidance of intimacy altogether [98].

One of the most significant concerns in this context is fertility. While not every survivor experiences infertility, the uncertainty surrounding their reproductive potential is a persistent source of anxiety. Survivors often struggle with when or whether to disclose their fertility risks to current or future partners. This can create emotional distance, lead to relationship strain, or generate a sense of guilt and inadequacy, even in stable partnerships. At the same time, some individuals report that openly navigating these challenges with a partner can strengthen mutual understanding and deepen the bond [99,100].

Sexual function is another area frequently affected. Cancer treatments—especially pelvic irradiation, certain chemotherapies, or hormone-disruptive protocols—can lead to persistent sexual difficulties, including discomfort, reduced libido, or difficulty with arousal and satisfaction. These issues are often compounded by psychological factors, such as the fear of rejection or shame, and may go unaddressed unless specifically assessed during survivorship care [83,99].

The impact of these challenges is not uniform. Individual experiences vary depending on the cancer type, treatment received, age at diagnosis, and available psychosocial support. However, the common denominator across studies is the need for integrated care that acknowledges the importance of sexuality and intimacy in long-term well-being. Guidelines increasingly recommend that fertility counseling, body image support, and relationship-focused interventions become standard components of survivorship care, starting in adolescence and extending into early adulthood [83].

Peer relationships are essential during adolescence, providing emotional support, shared experiences, and a sense of belonging. However, survivors of childhood cancer often struggle to socially reconnect after treatment. Prolonged absences from school, persistent fatigue, and visible reminders of illness—such as scars or medical devices—can lead to a persistent feeling of being “out of sync” with one’s peers. A systematic review by Peikert et al. confirmed that survivors frequently report emotional distress and difficulty rebuilding peer networks, emphasizing the long-lasting social impact of disrupted peer engagement [101].

Well-designed peer support programs can meaningfully counteract these challenges. Hemming et al., in their systematic review, found that interventions involving trained peer mentors, whether conducted face-to-face or virtually, effectively reduce loneliness, decrease depressive symptoms, and improve emotional resilience. These positive outcomes were most significant when the programs were embedded within school or clinical settings and supported by mental health professionals [80].

The practical implementation of peer support often combines structured mentorship with educational components for classmates and school personnel. Programs that integrate peer education with guided reintegration planning have been shown to improve emotional well-being, strengthen peer acceptance, and enhance classroom engagement among survivors. Educational activities aimed at increasing classmates’ understanding of cancer survivorship can foster empathy, reduce stigma, and contribute to a more inclusive and welcoming school environment during re-entry [102].

Still, the absence of structured peer engagement poses significant psychosocial risks. A review of models in Jamaica and Kenya highlighted that survivors without active peer support report higher levels of anxiety, depression, and diminished quality of life; these symptoms are frequently driven by internalized stigma, the fear of disclosure, or the mistrust of social interactions, factors that often go undetected unless explicitly addressed [80,101].

Lastly, teacher attitudes and preparedness play a crucial role in supporting peer reintegration. A quality improvement study by Klein et al. showed that when school personnel are educated and trained to manage returning students with a cancer history, covering academic, social–emotional, and medical needs, the transition is smoother. Teachers reported feeling more equipped, and students experienced fewer incidents of exclusion or misunderstanding [103].

No Polish studies have yet specifically examined romantic relationships or sexual health among adolescent survivors of childhood cancer. While data from adult-onset cancer survivors highlight issues like the decreased sexual quality of life, these findings are not directly transferable to a younger demographic. This gap underscores the critical need for age-appropriate national research to assess and support the intimate and relational needs of adolescent survivors.

#### 3.2.6. Transitions to Independence

Childhood cancer survivors often face significant hurdles as they transition into adult roles, such as entering the workforce, obtaining a driver’s license, and managing independent living. A meta-analysis of 93 cohorts reported that about 66% of survivors were employed in adulthood; however, employment rates varied significantly by diagnosis and treatment intensity: survivors of CNS tumors had a notably lower workforce participation (around 51%) compared to their peers who had less intensive treatments (approx. 80%). Furthermore, data from the CCSS indicate that adult survivors were nearly twice as likely to experience unemployment or to hold lower-skilled positions than sibling controls, with cranial irradiation, female sex, and a younger age at diagnosis being significant risk factors [104,105].

Achieving independent mobility—especially obtaining a driver’s license—is another critical milestone. Survivors often experience delays in this area due to cognitive impairments, visual–motor deficits, fatigue, and frequent medical appointments during adolescence. These factors can reduce their confidence and readiness for driving. As a result, the lack of transportation frequently emerges as a barrier to work, higher education, and social integration. Some survivors report long-term dependency on family members or public transport, which may contribute to social isolation [106].

Daily living tasks, such as managing personal finances, attending appointments independently, or maintaining self-care routines, also present difficulties, particularly in survivors with executive functioning deficits or chronic fatigue. Survivors often describe challenges in organizing their lives, meeting deadlines, or maintaining motivation. However, those who utilize structured routines, assistive technologies, or receive guidance from trained professionals report a better adaptation to independent living. Interventions such as occupational therapy, life skills workshops, and self-management training can be instrumental in supporting this transition [101].

Polish survivors frequently remain dependent on family support well into early adulthood, in part due to the limited vocational or life-skills training post-treatment. A 2017 national survey among all pediatric oncology departments in Poland highlighted that structured transition services are lacking: as many as 11 of 17 centers provided only a one-time, single transfer to adult care, with no dedicated curriculum or support system to foster young adults’ readiness for independence. This one-off handover often leaves survivors unprepared for adult healthcare systems, employment, or financial autonomy. Establishing dedicated transition programs, potentially including coordinated vocational counseling, progressive self-care education, and multidisciplinary AYA clinics, could significantly strengthen survivors’ autonomy and employment readiness [107].

### 3.3. The Need for a Biopsychosocial Approach

The comprehensive analysis presented in this review underscores the critical necessity of adopting a biopsychosocial model in the long-term care of childhood cancer survivors. The traditional biomedical model, while instrumental in acute oncological treatment, falls short in addressing the multidimensional late effects that emerge as survivors transition through adolescence into adulthood. These effects are not merely residual consequences of treatment but manifest in complex interactions between biological sequelae, psychological adaptation, and social functioning.

Biological challenges such as endocrine dysfunction, neurocognitive deficits, and chronic fatigue directly impede academic progress, employment opportunities, and independent living. These medical complications often co-occur with, or precipitate, psychological distress, manifesting as anxiety, depression, or post-traumatic stress symptoms. Simultaneously, the survivor’s altered developmental trajectory frequently disrupts normative social integration, romantic relationship building, and the achievement of adult autonomy. Consequently, siloed interventions targeting isolated medical or psychological symptoms are insufficient.

A truly effective long-term care framework must therefore integrate medical surveillance with psychological support and social reintegration strategies. Survivorship guidelines developed by groups such as the IGHG and COG increasingly endorse this multidimensional approach, recommending periodic psychosocial screening, neurocognitive assessments, and tailored interventions spanning health education, peer mentorship, and vocational support. Interdisciplinary collaboration is essential: oncologists, psychologists, social workers, educators, and rehabilitation specialists must coordinate efforts to deliver personalized, age-appropriate services that evolve with the survivor’s developmental stage.

In the Polish context, the absence of standardized national guidelines and fragmented follow-up protocols further highlights the urgency of adopting a biopsychosocial lens. Establishing institutional pathways for integrated care—including training clinicians in survivorship principles, developing school liaison roles, and embedding psychological screening tools into pediatric oncology follow-up—could bridge existing care gaps. Without such reform, many survivors risk falling through the cracks of the healthcare and social systems.

## 4. Limitations

Despite the methodological rigor employed in conducting this quasi-systematic review, several limitations must be acknowledged that may influence the comprehensiveness, generalizability, and interpretability of the findings.

As a quasi-systematic review, the methodology did not adhere strictly to all components of a fully systematic review (e.g., PROSPERO registration, exhaustive dual-reviewer data extraction across all stages). While this design provided flexibility in incorporating diverse sources—including gray literature and context-specific reports—it may have introduced a degree of selection bias due to less standardized screening procedures in some stages.

The reviewed literature displayed considerable heterogeneity in terms of study populations (e.g., age ranges, types of cancer, time since treatment), healthcare settings (e.g., country-specific systems), and outcome measures (e.g., different instruments assessing quality of life, psychological well-being, or social functioning). This diversity limited the potential for a direct comparison or meta-analytic synthesis and necessitated a narrative rather than a quantitative approach to data integration.

Although this review aimed to provide insights relevant to the Polish context, the current literature is heavily dominated by data from high-income Western countries such as the United States, Canada, and Western Europe. These models are often embedded in well-resourced healthcare systems and may not fully reflect the structural, financial, or cultural realities of Poland and other Central and Eastern European nations. As such, international findings should be interpreted with caution, and future efforts should prioritize regional studies and the context-sensitive adaptation of survivorship strategies.

The search strategy was limited to publications in English and Polish, which may have excluded relevant studies published in other languages. Furthermore, as with most literature reviews, there is an inherent risk of publication bias—i.e., the tendency for studies with positive or significant results to be more likely published than those with null or negative findings. This may have led to an overestimation of the effectiveness of certain survivorship interventions or the underreporting of challenges faced by survivors.

While gray literature sources were included to enrich the contextual understanding—particularly regarding gaps in the Polish healthcare system—such sources often lack rigorous peer review. The quality, transparency, and reproducibility of findings in these documents may vary, and their interpretation must be approached with caution.

Although this review encompassed studies published between 2010 and 2025, some potentially relevant older foundational works may have been omitted. Additionally, the literature published after the conclusion of the search period (April 2025) was not included, which may impact the currency of findings in a rapidly evolving field such as survivorship care.

Despite efforts to minimize the bias through dual screening and consensus resolution, the quasi-systematic nature of this review design inherently increases the risk of subjective judgment in the study selection, data extraction, and interpretation. The absence of third-party adjudication in all stages may have influenced the inclusion or weighting of certain studies or themes.

## 5. Conclusions and Future Directions

Childhood cancer survivorship is no longer defined solely by remission or cure but by the survivor’s capacity to thrive across all dimensions of life. This review illuminates the scope of biopsychosocial late effects experienced by adolescent survivors, ranging from organ-specific sequelae to disruptions in mental health, education, peer relationships, employment, and independent living. While advances in treatment have extended survival, they have also created a new category of complex, long-term patients whose needs evolve over time.

Evidence shows that adolescence is a critical window for intervention, as developmental milestones coincide with the manifestation of many late effects. Survivors require long-term monitoring using validated tools, such as the PHQ-9, GAD-7, PTSD-RI, and BRIEF, and interventions tailored to developmental tasks including peer integration, sexual health, and vocational planning. Fragmented or medically limited follow-up systems risk neglecting these critical aspects, particularly in countries like Poland, where no unified model currently exists.

The findings reinforce the need for coordinated multidisciplinary follow-up care grounded in the biopsychosocial model. Such care must be proactive, developmentally informed, and sustained well into young adulthood. It should incorporate medical surveillance, psychological support, educational liaisons, and social reintegration programs as standard elements of survivorship care.

Addressing these systemic gaps in Poland will require the development of a cohesive survivorship framework that is both evidence-informed and adapted to the specific characteristics of the national healthcare system. Drawing on the international experience, such a framework should prioritize the continuity of care, institutional coordination, and the integration of psychosocial services throughout the survivorship trajectory. Embedding long-term follow-up into national policy, improving access to age-appropriate psychological care, and fostering interdisciplinary training can provide a foundation for sustainable reform. An initial implementation within academic or tertiary-care centers may help evaluate the feasibility and support broader scalability.

Only by acknowledging and addressing the full spectrum of survivors’ needs can we ensure that childhood cancer does not define their future but becomes one part of a resilient and supported life trajectory. By addressing adolescence as a distinct developmental stage and including regional insights, this review offers a unique contribution to the literature on psychosocial survivorship after childhood cancer.

Incorporating therapeutic approaches into survivorship care may significantly enhance long-term psychosocial outcomes for adolescent cancer survivors. Evidence-based methods such as cognitive-behavioral therapy (CBT) have shown efficacy in reducing symptoms of anxiety and depression. Peer support interventions can help restore social connectedness, while school-based programs and structured transition services support reintegration and promote greater independence in early adulthood.

## Figures and Tables

**Table 1:** An overview of organ-specific long-term biological complications associated with pediatric cancer therapy: a system-based classification with underlying pathophysiological mechanisms.

Complication Group	Typically Encompassed Conditions	Source
Cardiotoxicity	Dilated cardiomyopathy, arrhythmia, congestive heart failure, autoimmune myocarditis, autoimmune pericarditis, vascular diseases, including coronary, coronary, large vessels, and RIHD.	[17,18,19,20,21,22]
Nephrotoxicity and renal dysfunction	Acute and chronic kidney disease, glomerulopathies, tubulointerstitial nephritis, tubular injury, Fanconi syndrome, renal tubular acidosis, and nephrogenic diabetes insipidus, and electrolyte disturbances.	[23,24,25,26,27,28,29]
Hepatotoxicity	SOS, hepatic fibrosis, and steatosis.	[30,31,32,33,34,35,36]
Endocrine disorders	Hypothalamic-pituitary axis impairment hyperprolactinemia, central diabetes insipidus, thyroid dysfunction, gonadal failure, infertility, and ACTH deficiency.	[37,38,39,40]
Growth and skeletal development disorders	Impaired longitudinal growth, osteopenia, skeletal deformities, and AVN.	[41,42]
Neurotoxicity	Epilepsy, encephalitis, ischemic stroke, encephalopathy, posterior fossa syndrome, chronic headaches, peripheral neuropathy, paresis and paralysis, ataxia, hearing, vision, and other sensory disorders.	[43,44,45,46,47,48,49]
Immunosuppression and immune disorders	Prolonged B- and T-cell depletion, increased susceptibility to infections.	[50,51,52]
SMNs	Therapy-related leukemias, sarcomas, and radiation-induced solid tumors.	[53,54,55]

Abbreviations: RIHD, radiation-induced heart disease; SOS, sinusoidal obstruction syndrome; ACTH, adrenocorticotropic hormone; and AVN, avascular necrosis.

**Table 2:** Most common mental health disturbances among adolescent childhood cancer survivors (13–19 years).

Clinical Parameter	Prevalence	Relative Risk/OR (95% CI) vs. Age-Matched Peers	Source
Global distress (BSI-18 ≥ 63)	13.0% to 25.0% (increase over 2.4 years)	no data	[62]
Anxiety	7.4%	OR 2.00 (1.17–3.43)	[61]
Depression	11.7%	OR 1.55 (1.04–2.30)	[61]
Post-traumatic stress disorder (PTSD)	8.6%	RR 2.36 (1.37–4.06)	[59]
Suicidal ideation	9.0%	RR 1.67 (1.38–2.02)	[64]
Completed suicide	0.3%	RR 1.50 (0.63–3.62)—not significant	[64]
Clinically significant fear of cancer recurrence	16.6%	no data	[60]
Moderate/high cancer-related worry	38.0%	no data	[65]

## Data Availability

Data sharing is not applicable.

## Supplementary Materials

The following supporting information can be downloaded at: https://www.mdpi.com/article/10.3390/children12080980/s1, Table S1: An extended overview of organ-specific long-term biological complications associated with pediatric cancer therapy: a system-based classification with underlying pathophysiological mechanisms.

## Abbreviations

The following abbreviations are used in this manuscript:

LTFU	Long-Term Follow-Up
CINAHL	Cumulative Index to Nursing and Allied Health Literature
PBL	Polska Bibliografia Lekarska
PTSD	Post-Traumatic Stress Disorder
IGHG	International Late Effects of Childhood Cancer Guideline Harmonization Group
COG	Children’s Oncology Group
CNS	Central Nervous System
CCSS	Childhood Cancer Survivor Study
FCR	Fear of Cancer Recurrence
ALL	Acute Lymphoblastic Leukemia
DCCSS-LATER	Dutch Childhood Cancer Survivor Study-Late Effects After Childhood Cancer
GAD-7	Generalized Anxiety Disorder-7
AYA	Adolescent and Young Adult
PHQ-9	Patient Health Questionnaire-9
BRIEF	Behavior Rating Inventory of Executive Function
GEC	Global Executive Composite
BRI	Behavioral Regulation Index
ADHD	Attention Deficit Hyperactivity Disorder
IEP	Individualized Education Plan
RIHD	Radiation-Induced Heart Disease
SOS	Sinusoidal Obstruction Syndrome
ACTH	Adrenocorticotropic Hormone
AVN	Avascular Necrosis
CBT	Cognitive–Behavioral Therapy
