# Supplementary material for: Adolescent Survivors of Childhood Cancer: Biopsychosocial Challenges and the Transition from Survival to Quality of Life
Source: Children (Basel). 2025 Jul 25;12(8):980. doi: 10.3390/children12080980 (PMC12384235; doi:10.3390/children12080980)
Supplement: Supplementary file 1 [file children-12-00980-s001.zip › children-3760539-supplementary.pdf]

Table S1. An extended overview of organ-specific long-term biological complications associated with pediatric cancer therapy: a system-based classification with underlying pathophysiological mechanisms.

Complication group	Pathomechanism			Source
	Chemotherapy	Radiotherapy	Immunotherapy	
Cardiotoxicity	Action on TOP2, isoenzyme TOP2B; direct inhibition of the RYR2 and SERCA2A; effect on transmembrane transport of Ca^{2+}; binding of the F1F0 proton pump in the mitochondria of cardiomyocytes; toxic effect of CBR3 metabolite with SNP (rs1056892); dysfunction of proteins of the ABCC1; oxidative metabolism and ROS - SOD2 and GSTT1, GSTM1 disorders; polymorphism of the CELF4 gene family; influence on histamine H1 and H2 receptors by CrEL; activation of the ER stress response; myocyte vacuolization; myofibrillar lysis; promotion of cytokine synthesis and apoptosis; direct damage to the endothelium, thrombosis, inflammation, and vasoconstriction;	Oxidative stress and ROS production; direct DNA damage; disruption of ncRNA homeostasis activation of apoptotic mechanisms; protein damage and degradation; release of calcium from the endoplasmic reticulum; increased concentration of TGF-β1, IL-1, IL-6, PDGF, MCF, and NF-kB; promotion of interstitial fibrosis; metabolic changes in cardiomyocytes (transition from fatty acid oxidation to glycolysis); endothelial damage and dysfunction; promotion of thrombosis; impaired production of vasodilators (e.g., NO)) and vasoconstriction; autonomic dysfunction.*	CRS - increased concentrations of IL-6, IL-1, IFN-γ and TNF-α; increased expression of IL-2 receptors; impaired cardiomyocyte metabolism; impaired repair mechanisms; initiation of apoptotic mechanisms; autoimmune reaction - mainly cardiolipin antigens; antiangiogenic effect; coagulopathy; leakage; vascular; microcirculation dysfunction; hypotension; damage to the vascular endothelium.* <br>*Lack of complete knowledge and explanation due to lack of long-term clinical monitoring.	[17–22]

	autoimmune response increased susceptibility due to acquired and congenital pathologies—HTN, hereditary hemochromatosis	*For every 1 Gy increase in the average dose to the right coronary artery, the risk of CAD increased by 28.7%.		
Nephrotoxicity and renal dysfunction	Production of large amounts of chloroacetaldehyde (highly toxic to epithelial cells); induction of glycosuria, phosphaturia, and aminoaciduria, albuminuria, and proteinuria; impaired reabsorption of phosphate and magnesium; reduced excretion of Tamm-Horsfall protein; accumulation of toxic platinum metabolites; mitochondrial damage; reduction of sulfhydryl groups in mitochondrial proteins and calcium uptake; DNA and RNA damage; precipitation of methotrexate and its less soluble metabolites in acidic urine.	Direct damage to nephrons and renal tubules; renal artery stenosis; increased expression of proinflammatory and profibrotic cytokines promoting fibrosis.<br><br>*radiation doses >25 Gy have been associated with a decrease in kidney function	Non-specific nephrotoxicity; CRS and pro-inflammatory environment generation, sepsis, and tumor lysis syndrome; coagulation disorders, thrombocytopenia, thrombotic microangiopathy; loss of tolerance to self-antigens of the kidneys; off-target effects on tubular cells overexpressing PD-L1; hypocalcemia associated with PD1 blockade renal vasculitis; CAR-T conditioning - cytapheresis and lymphodepleting chemotherapy (often a combination of cyclophosphamide and fludarabine); CAR-T cell infiltration into the renal parenchyma.	[23–29]
Hepatotoxicity	Production of reactive metabolites	Damage to small hepatic vessels;	Expression of CTLA-4 on regulatory T cells	[30–36]

	generated by phase I oxidation reactions; changes in mitochondrial function; induction of inflammation and hyperplasia; immune responses; direct toxicity to healthy liver tissue (apoptosis, necrosis, and necroptosis); individual sensitivity conditioned by genetic variability.	edema of endothelial cells; stenosis of hepatic veins and partial or complete obstruction of small veins in the hepatic parenchyma; hypoxic death of hepatocytes, necrosis, and liver atrophy.* *The risk of radiation-induced SOS was estimated at ≤5% at whole liver doses ≤30 Gy and ≤28 Gy (at 2 Gy per fraction).	and PD-L1 on hepatocytes and other non-parenchymal cells, such as stellate and Kupffer cells; clonal expansion of cytotoxic, Th1 and Th17 cells and suppression of Treg cells; dominance of proinflammatory mediators in inflammatory homeostasis; innate sensitivity of immune system cells.	
Endocrine disorders	Direct toxic damage to endocrine organs; apoptosis of reproductive cells; DNA damage, changes in DNA methylation and histone modification oxidative stress, induction of inflammation.	Damage to hormone-synthesizing cells; fibrosis of endocrine organs by mediators.* *The most sensitive hormone is GH, followed by LH, FSH, TSH, and ACTH.	Mechanism is not fully understood; CRS; autoimmune reactions; cellular and humoral immune response disorders; cross-reactions; chemokine stimulation; genetic predisposition.	[37–40]
Growth and skeletal development disorders	Impaired DNA synthesis in osteoblasts; changes secondary to CUN damage and growth hormone deficiency; changes secondary to gonadal damage and sex hormone deficiency.	Damage to cells synthesizing growth hormone; growth hormone is the most sensitive to damage from radiotherapy among pituitary hormones.* *Brain RT at a dose above 18 Gy.	Secondary changes to pituitary damage and growth hormone deficiency; CRS.	[41,42]

Neurotoxicity	Toxic effect on cells during DNA replication; damage to the neuronal cytoskeleton; neurogenesis disorders; microglia activation; inflammatory process with increased cytokine and chemokine concentrations; myelination disorders; oxidative stress; neurotransmitter imbalance; reduced blood vessel density.	Direct damage to nerve cells, progenitor cells, and stromal cells; decreased blood vessel density; radiation-induced inflammation; damage to oligodendrocytes and white matter necrosis; insufficient myelination; oxidative stress.	CRS; lack of self-tolerance due to the involvement of B and T cell-mediated mechanisms (autoantibodies); cells damage to astrocytes; osmotic dysregulation activation of endothelial cells; disruption of blood-brain barrier selectivity.	[43–49]
Immunosuppression and immune disorders	Myelosuppression and a decrease in the global number of leukocytes (e.g., thymopoiesis deficiencies due to a lack of T lymphocyte progenitors); disorders of maturation and function of T, NK, and B lymphocytes; disorders of maturation and function of DC; increase in suppressor cells – MDSC; arginine depletion; ROS production; decreased immunoglobulin production; impaired immune response;	Destruction of bone marrow stem cells; ICD, releasing DAMPs (e.g., HMGB1); induction of ROS production; damage to innate immune barriers (e.g., intestinal mucosa).	CRS; autoimmune disorders; excessive T-cell response; suppressive TME (IL-10, TGF-β, MDSC, Treg).	[50–52]

	limited response to antigens contained in vaccines – immune memory disorders; damage to innate immune barriers (e.g., intestinal mucosa).			
SMNs	Induction of mutations and deletions (mainly chromosomes 5 and 7), leading to MDS/AML; DNA breaks and translocations (e.g., 11q23 - after etoposide).	Induction of point mutations; large genome damage; translocations.* *Radiotherapy is associated with the highest risk of SMNs.	Potentially: risk of mutagenic insertions; CAR-T against CD19 sometimes cause B-ALL to transform into AML or vice versa (immune escape).	[53–55]

Abbreviations: TOP2, topoisomerase II; RYR2, ryanodine receptor 2; SERCA2A, Ca+2-ATPase; Ca^{2+}, calcium ions; CBR3, carbonyl reductase; ABCC1, C subfamily member 1 of the ATP-binding cassette; ROS, reactive oxygen species; SOD2, superoxide dismutase II; GST, glutathione S-transferase; CELF4, CUGBP Elav-like 4; CrEL, Cremophor EL; ER, endoplasmic reticulum; HTN, hypertension; ncRNA, non-coding RNA; IL-1, interleukin-1; IL-2, interleukin-2; IL-6, interleukin-6; IL-10, interleukin-10; PDGF, platelet-derived growth factor; MCF, monocyte chemotactic factor; NF-kB, nuclear factor kappa B subunit 1; NO, nitric oxide; CAD, coronary artery disease; CRS, Cytokine release syndrome; IFN-γ, interferon gamma; TNF-α, tumor necrosis factor alpha; RIHD, radiation-induced heart disease; CAR-T, Chimeric Antigen Receptor T-cell; PD-L1, programmed death-ligand 1; PD1, programmed cell death protein 1; CLTA4, cytotoxic T cell antigen 4; Th-1, T-helper 1; Th-17, T-helper 17; Treg, Regulatory T cells; SOS, Sinusoidal obstruction syndrome; GH, growth hormone; LH, luteinizing hormone, FSH, follicle-stimulating hormone, TSH, thyroid-stimulating hormone, and ACTH, adrenocorticotropic hormone; AVN, avascular necrosis; DC, dendritic cells; MDSC, Myeloid-derived suppressor cells; ICD, immunogenic cell death; DAMPS, damage-associated molecular patterns; HMGB1, High-mobility group box 1; TME, tumor microenvironment; TGF-β, Transforming growth factor beta; SMNs, Subsequent malignant neoplasms; MDS, Myelodysplastic Syndromes; AML, acute myeloid leukemia; ALL, acute lymphoblastic leukemia.
